# Recruiting to a large-scale physical activity randomised controlled trial – experiences with the gift of hindsight

**DOI:** 10.1186/s13063-016-1229-0

**Published:** 2016-02-24

**Authors:** Robert J. Copeland, Kimberley Horspool, Liam Humphreys, Emma Scott, on behalf of the Booster trial team

**Affiliations:** Centre for Sport and Exercise Science, Sheffield Hallam University, Collegiate Campus, Sheffield, S10 2BP UK; School of Health and Related Research, University of Sheffield, Regent Court, 30 Regent Street, Sheffield, S1 4DA UK; Warwick Medical School, University of Warwick, Coventry, CV4 7AL UK

**Keywords:** Recruitment, Physical activity, Mail-outs, Booster trial, Behaviour maintenance

## Abstract

**Background:**

Recruitment issues continue to impact a large number of trials. Sharing recruitment information is vital to supporting researchers to accurately predict recruitment and to manage the risk of poor recruitment during study design and implementation. The purpose of this article is to build on the knowledge available to researchers on recruiting to community-based trials.

**Methods:**

A critical commentary of the recruitment challenges encountered during the Booster Study, a randomised controlled trial in which researchers investigated the effectiveness of a motivational interviewing style intervention on the maintenance of physical activity. An overview of recruitment is provided, as well as strategies employed to recruit prospective participants and possible barriers to recruitment.

**Results:**

Two hundred eighty-two people, 47 % of the original target, were recruited through mail-outs, with secondary recruitment pathways yielding no additional participants. The research team encountered problems with recontacting interested participants and providing study materials in non-English languages. A lower response rate to the mail-out and a greater number of non-contactable participants in the full study than in the pilot study resulted in a smaller pool of eligible participants from the brief intervention eligible for recruitment into the randomised controlled trial.

**Conclusions:**

Despite using widely accepted recruitment strategies and incorporating new recruitment tactics in response to challenges, the Booster Study investigators failed to randomise a sufficient number of participants. Recruitment in trials of community-based behavioural interventions may have different challenges than trials based on clinical or primary care pathways. Specific challenges posed by the complexity of the study design and problems with staffing and resources were exacerbated by the need to revise upwards the number of mailed invitations as a result of the pilot study. Researchers should ensure study design facilitates recruitment and consider the implications of changing recruitment on the operational aspects of the trial. Where possible, the impact of new strategies should be measured, and recruitment successes and challenges should be shared with those planning similar studies.

ISRCTN56495859 (registered on 12 February 2009); NCT00836459 (registered on 3 February 2009).

## Background

Many randomised controlled trials (RCTs) suffer from poor recruitment. Up to two-thirds of research studies published in *The Lancet* and *BMJ* in 2000 and 2001 failed to meet recruitment targets or required an extension to do so [1]. More recently, researchers examining studies funded by the National Institute for Health Research (NIHR) Health Technology Assessment (HTA) Programme and the U.K. Medical Research Council (MRC) suggested that 31–55 % recruited to target and 45–54 % required an extension of some kind [2, 3]. Poor recruitment can impact external validity [4], statistical power [5] and likelihood of publication [6]. The financial burden passed on to research funders as a consequence of study overrun is likely to be significant and could influence funders’ decisions to invest in future research [7]. Therefore, reducing the amount of studies which fail to recruit to time and target is critical.

Encouragingly, a number of authors have reported on trial recruitment within health research [7–9]. A limitation of this evidence, however, is the reliance on ‘mock’ trials, where recruitment to non-RCTs is conducted. The systematic techniques for assisting recruitment to trials (START) project [10] marks a methodological change in how researchers investigate recruitment. While START’s nested trials are being undertaken, it is important for other researchers to provide a transparent commentary on recruitment issues, particularly in complex trials or those targeting specific populations, thus expanding the body of evidence available to researchers planning studies.

Community-based trials make up only a small proportion of studies represented in recent reviews (e.g., 7 of 122 [3]). Sully et al. [2] were unable to comment on trial setting as a factor in successful recruitment, as the number of community-based studies available was too small to draw statistically reliable conclusions. They were, however, able to confirm that recruitment difficulties in community settings are consistent with those across other settings, with unsuccessful recruitment in 47 % of studies. Recruitment to community-based RCTs is likely to involve challenges different from those in clinical settings which use clear care pathways. Although some evidence exists on the recruitment to pragmatic studies from community-dwelling populations [11, 12], the low rate of recruitment to such studies requires further exploration. This is particularly the case regarding studies in which investigators seek to recruit individuals to health behaviour change interventions, a field which is under-represented in the review evidence.

With this in mind, in this article we present a transparent examination of the recruitment experience from the Booster Study, which was funded by the NIHR HTA Programme (project reference 07/25/02) and approved by Sheffield Research Ethics Committee (reference 08/H1308/270). Details of the study design, eligibility criteria, interventions and outcome measures have been published previously [13]. The insights provided herein have been informed by data derived from field notes, trial records and research staff consultations [e.g., responses to open-ended questions sent via email from the study manager to the research assistants (RAs)] and therefore provide valuable information on the day-to-day management and delivery of complex interventions related to physical activity, which is rarely documented in the extant literature. The study was community-based, recruiting healthy individuals from deprived socio-demographic urban areas, into a physical activity behaviour change intervention. Understanding the recruitment issues in this study could support other researchers who are also planning to conduct research in challenging settings, with non-medical interventions.

## Methods

### Overview of the Booster Study

The Booster Study was an RCT in which researchers investigated the effectiveness of different intensities of booster intervention to maintain physical activity in previously sedentary adults who had recently become more physically active as a result of a brief intervention. The brief intervention was use of an interactive DVD to promote physical activity and engender behaviour change, which was based upon the principles of motivational interviewing [14] and informed by National Institute for Health and Care Excellence guidance [15, 16]. Eligible participants were randomised to one of three groups: booster sessions delivered (1) face to face, (2) over the telephone, or (3) no booster sessions (usual care).

### Recruitment pathway

The Booster Study had a complex, two-stage recruitment process which included a number of ‘passive’ and ‘active’ recruitment strategies [17]. Potential participants were first recruited to the brief intervention, largely via passive methods (e.g., mail-outs, posters, flyers, press releases), and after 3 months those who had successfully increased their physical activity by at least 30 minutes per week were recruited into the RCT with adoption of an active recruitment approach (e.g., telephone contact via researchers). An internal pilot and feasibility study was conducted before the full trial. This enabled the research team to assess the suitability of the sample size and to test the recruitment pathway [18]. In the pilot study, the researchers aimed to recruit 60 participants into the RCT over a 2-month period (November and December 2009). The recruitment target for the main trial was 600 participants (including pilot study participants) over 18 months (June 2010 to November 2011). Participation from start to finish was estimated at 13 months; a further breakdown of the timescales for recruitment and the main trial research activities is outlined in Table 1.

**Table 1:** Booster Study research activities and timescale

	−4 months	−3 months	−2 months	−1 month	−1 week	Main trial start	1 month	2 months	3 months	9 months
Invitation letter sent	✔									
Interested individuals contact and screened for brief intervention eligibility		✔								
Eligible participants are sent brief intervention		✔								
Participant contacted for DVD use assessment			✔	✔						
Participant contacted and screened for main trial eligibility					✔					
Face-to-face appointment, consent, baseline assessment and randomisation						✔				
Participant receives motivational interviewing (excludes control group)							✔	✔		
Face-to-face follow-up appointment									✔	
Face-to-face follow-up appointment										✔

#### Recruitment to the brief intervention – primary strategy

The primary recruitment strategy was a personally addressed invitation letter printed on National Health Service (NHS)–headed paper and signed by the director of public health of the local NHS primary care trust (PCT). The letter invited individuals to become involved in a large physical activity study whose aim was to help people become physically active and stay physically active, with the first stage being to receive the free DVD. Invitation letters were sent to all community-dwelling adults aged 40–65 years living in deprived neighbourhoods in the city of Sheffield, UK. A free-post reply card was included with the letter, which individuals were required to complete and return. It included telephone and email contact details, preferred contact time, preferred language of DVD and whether the individual needed support to access a DVD player.

#### Recruitment to the brief intervention – additional strategies

To maximise the pool of participants available for later recruitment into the RCT, the PCT mail-out was complemented by a range of other strategies. All recruitment packs distributed through these routes were non-personalised, but reply cards were coded to ensure that the research team could identify the source of recruitment and costs could be reimbursed where appropriate.

1. *General practitioners*: General practices serving the target neighbourhoods were contacted by the research team in August 2010. The initial letter included a one-page summary of the study and asked practices to distribute recruitment packs to potentially eligible patients during consultations. General practitioners (GPs) and practice nurses were asked to consider all 40–65-year-olds whom they believed to be sedentary and attending the practice as potentially eligible. Any patients subsequently returning the free-post reply card would have their eligibility confirmed by the research team. Practices were approached a second time via the local primary care research network (PCRN) in April 2011, with information provided in the standard PCRN format.
2. *Enhanced public health programme leads*: At the time of the study, there were 15 enhanced public health programme (EPHP) areas in Sheffield, which covered the majority of the target neighbourhoods. The aim of the EPHP was to address health inequalities in the areas of the city with the poorest health by working with local communities to promote healthy lifestyles; improve access to services for prevention, treatment and care; and tackle the root causes of ill health. The study manager presented the Booster Study at the EPHP leads meeting in October 2009 and made two requests: (a) for information about physical activity opportunities in their EPHP area, particularly small, independent activities which the research team might otherwise remain unaware of; and (b) assistance making links with community leaders or organisations that could help promote the Booster Study within the typically hard-to-reach communities being targeted. The study manager then contacted each EPHP lead individually shortly before invitation letters were sent out in their area to reiterate these requests. The EPHP leads were reimbursed for their time spent on these activities.
3. *Health trainers and health champions*: Sheffield has a network of health trainers (paid) and health champions (volunteers) who support people to adopt healthier lifestyles. These services were located within all target neighbourhoods. It was agreed that the health trainers and health champions would distribute recruitment packs and later encourage potential participants to engage in the RCT. This information was communicated to the health trainers and health champions by the network co-ordinator before the study manager visited the host sites and organisations.
4. *One-stop health shops*: Across the City of Sheffield, there are a number of ‘one-stop health shops’ that offer access to a wide range of community-based health services, such as smoking cessation and diabetes self-management support. These services were contacted directly by post or email as the study was rolled out in their neighbourhoods. A number of these services agreed to distribute recruitment packs to clients who expressed an interest in becoming more active.
5. *Local community groups*: Community group leaders are often key people in the local community. A range of different community groups based in the target neighbourhoods were identified, including churches, mosques, the South Asian taxi drivers health initiative, the Somali Community Health Project and the Roshni South Asian Women’s Resource Centre. In August 2010, letters were sent to religious and group leaders introducing the Booster Study and inviting them to contact the team if they thought their congregation or group might be interested in further information.
6. *Press releases*: The various recruitment routes were accompanied by two press releases (January and June 2010) which aimed to raise awareness of the study but did not offer a direct route to recruitment.
7. *Promotional materials*: Posters were displayed in venues where recruitment was facilitated, such as GP surgeries and local community centres. The poster provided the telephone contact details of the research team and the Booster Study website address so that prospective participants could gather more information.

#### Screening for the brief intervention

Individuals who returned the reply card were contacted by telephone to complete a health screening and to assess their current physical activity using the Scottish Physical Activity Questionnaire [19]. Contact was attempted at the preferred times indicated on the reply cards, including evenings and weekends where possible, and multiple attempts were made, using voicemail facilities when available. Those who were classified as sedentary (i.e., not achieving at least 30 minutes of moderate physical activity on at least 5 days of the week) were sent the brief intervention (DVD and written information about local exercise opportunities) through the post. Participants with contraindications to exercise were recommended to seek medical advice before using the DVD.

#### Recruitment to the main intervention (RCT)

Individuals who received the brief intervention were recontacted after 3 months to assess their eligibility for the RCT. Those who reported an increase of at least 30 minutes of physical activity per week, compared with brief intervention screening, were deemed eligible and given verbal information about the RCT. They were then invited to book an initial appointment at a time and location convenient to them. They were offered a choice of 13 venues across the city with appointment times available both during the day and in the evening. Letters confirming appointments as well as the participant information sheet were sent by post in advance of this meeting. Text message reminders were sent 24 h before the appointment to reduce non-attendance; in the event of non-attendance, the individual was contacted and given the opportunity to rebook. Written informed consent was taken at this appointment before the completion of baseline assessments and randomisation into one of the three trial arms.

## Results

### Overview of recruitment

**Fig. 1:**
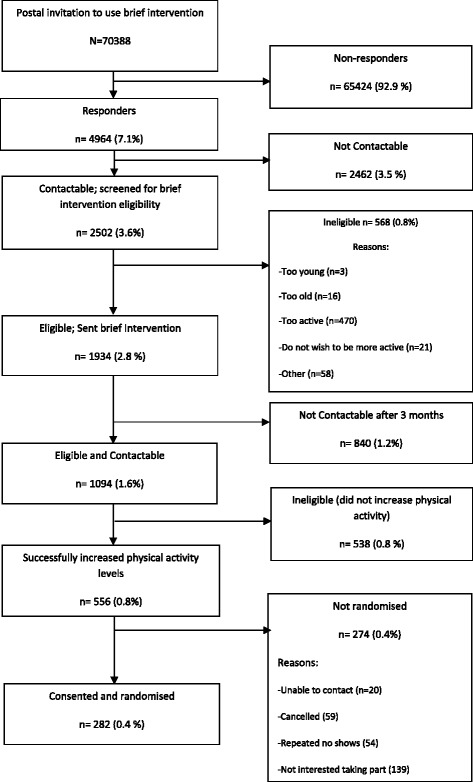
CONSORT (Consolidated Standards of Reporting Trials) diagram of overall recruitment

Two hundred eighty-two people were recruited into the RCT over a period of 24 months (November 2009 to November 2011), which equated to 47 % of the recruitment target of 600 participants. The Booster Study management team decided a funded extension to prolong recruitment would not represent value for money for the funding body, as an independent analysis by the Data Monitoring and Ethics Committee revealed a greater than anticipated standard deviation in the primary outcome measure which impacted the ability of the study to reliably answer the research question. The recruitment figures for the full trial (including the pilot study) are presented in Fig. 1.

### Recruitment stages

#### Recruitment to the brief intervention – primary strategy

Invitation letters were sent to 70,388 individuals. This figure was revised upwards from the initial estimation of 30,000 [13] as a result of the response and recruitment rates observed in the pilot study [18]. The overall response rate was 7.1 % (*n* = 4964), which is identical to that achieved by Forster et al. [11], whose study was also based in South Yorkshire, UK; however, it is substantially lower than the 28 % (*n* = 1439) achieved by Hardcastle et al. [20]. Among the responders, 2502 individuals completed the brief intervention screening; the research team was unable to contact the remaining 49.5 % (*n* = 2462). Participants eligible for the brief intervention (*n* = 1934) were sent the DVD. Reasons for ineligibility included being too young (aged <40 years; *n* = 3), too old (aged >65 years; *n* = 16), too active (self-reported physical activity at least 30 minutes on at least 5 of the last 7 days; *n* = 470), not wishing to be more active (*n* = 21) and other reasons (*n* = 58).

#### Recruitment to the brief intervention – additional strategies

1. *General practitioners*: Thirty-nine general practices were provided with information about the study, and six expressed an interest as a result of the initial contact from the research team. There was no further interest after the PCRN contact. The study manager attended each practice to give a detailed briefing about the Booster Study, which included a short presentation and discussion about the study that included the aims, study design, how to identify potentially eligible participants, and reimbursement for recruitment. A total of 305 coded recruitment packs were distributed to these practices. No coded reply cards were received by the research team, nor was data collected from the practices to determine how many packs were distributed. A further 18 practices agreed to display a poster providing information about the study that included the research team’s contact details.
2. *Enhanced public health programme leads*: Despite the good fit of the brief intervention with the remit of the EPHP, engagement from the EPHP leads was variable. Five leads provided information about local physical activity options, but only four of fifteen facilitated links to community leaders or organisations.
3. *Health trainers and health champions*: A total of 6 health trainers and 20 health champions were provided with information about the study. Recruitment packs were available at all seven host sites across the city. No reply cards were received. The number of packs given to clients is unknown.
4. *One-stop health shops*: Three one-stop health shops agreed to offer recruitment packs to clients. A total of 90 coded recruitment packs were distributed to these shops, but no reply cards were received. Again, the number of packs given to clients is unknown.
5. *Local community groups*: The study manager wrote to the 123 churches and 27 mosques located in the target neighbourhoods. Two churches responded and were visited by the study manager, who explained the study in more detail. One church also requested recruitment packs in case any members of the congregation were interested and were not able to find the original invitation letter from the PCT. This was an unexpected request, and as a result the packs were not coded, making them indistinguishable from the responses to the PCT invitation letters. A positive response was also received from a women’s group that was part of the Somali Community Health Project, although when the study manager visited the group to talk about the study, there appeared to be some confusion about what was being offered, with many women expecting the study manger to deliver an exercise class.

#### Possible barriers to recruitment

##### Non-contactable participants

Contacting potential participants was a problem throughout the study. Despite confirming addresses as current with the PCT the week before each mail-out, 1117 letters (1.6 %) were returned due to the addressee’s being unknown at that address. There were undoubtedly more letters which were not returned to sender, suggesting that a sizable minority of invitations were not received. Of the 4964 who did reply to the invitation, 2462 (49.6 %) were not contactable and a further 840 (16.9 %) were no longer contactable 3 months after receiving the brief intervention. Participants were termed *non-contactable* if the contact details provided by the participant were incorrect and telephone or email contact could not be made, or if three attempts at contact failed.

##### Language requests

There were 162 requests (3.3 %) for the brief intervention in a language other than English. The number of different languages requested was much greater than expected (*n* = 19). Thus, it was not possible to have the DVD translated to accommodate all of these requests. Letters were sent to those asking for the DVD in another language, explaining that the DVD was not available in the language requested and asking whether an English version would be acceptable. Seventy-nine people accepted the offer of an English version of the DVD, four declined and the remaining seventy-nine did not reply.

##### DVD player access

People who needed assistance accessing a DVD player to use the brief intervention DVD were offered the loan of a portable DVD player for 3 months, during the brief intervention phase of the study.

#### Recruitment to the main intervention (RCT)

One thousand ninety-four individuals (56.6 %) who received the brief intervention were contactable 3 months later for assessment of their eligibility for the RCT. Of these, 50.8 % had achieved an increase of 30 minutes of moderate-intensity physical activity per week and thus met the eligibility criteria. This was greater than the 40 % eligibility rate predicted [13], but it still meant that only 556 individuals were eligible for the RCT, already lower than the recruitment target (*n* = 600).

Numbers declined further when the opportunity to participate in the RCT was offered. The most common reasons for non-participation were not interested (25 %, *n* = 139) and cancelled or failed to attend a baseline appointment on more than one occasion (20.3 %, *n* = 113). Poor conversion from eligible to randomised participants was exacerbated by those who were non-contactable after a failure to attend (*n* = 20) the baseline assessment. Low conversion into support for physical activity has been observed elsewhere, including with other community-based interventions [21].

### Comparison of pilot study and full trial recruitment

Despite our conducting a pilot study to test recruitment, there were notable differences in recruitment between the pilot study and the full trial (Table 2). An unexpectedly lower response rate in the full trial than in the pilot study (7.1 % vs. 9.9 %) equated to 1970 fewer responses than anticipated. This was compounded by a much greater proportion of individuals who were non-contactable at brief intervention screening (49.5 % vs. 15.8 %) and a lower proportion eligible for the RCT (50.0 % vs. 78.8 %), leading to a decreased participant pool for the full trial.

**Table 2:** Comparison of pilot study and full trial recruitment

	Pilot study	Full trial
Response rate	9.9 %	7.1 %
Non-contactable for brief intervention screening	15.8 %	49.5 %
Too active for brief intervention	30.0 %	18.0 %
Non-contactable for main trial screening	43.4 %	44.7 %
Eligible for main trial	78.8 %	50.0 %
Consented and randomised	57.0 %	50.0 %

## Discussion

The Booster Study investigators successfully recruited 282 participants. This sample size is similar to samples in other public health studies [11], albeit markedly less than in some studies of physical activity in which nearly 950 participants were recruited [22]. The final sample size, however, is only one small part of the story, and the distillation process which yielded the 282 participants offers several points of interest.

### Consideration of the study design: did it facilitate recruitment?

Out of necessity, the Booster Study had a very complex recruitment pathway: The intervention was intended to help participants maintain recent increases in physical activity behaviour. Thus, a recruitment pool of potential participants needed to be created, as there was no existing way to easily identify and contact such people. In previous research, investigators have identified strategies and study designs which are thought to enhance RCT recruitment [23], one of which is a simple study design; however, the Booster Study could not be said to have this. Table 3 lists other features known to aid in recruitment to trials and highlights how the Booster Study researchers implemented 23 of 28 of these strategies. It can be argued that the context of the Booster Study meant that some features would be unlikely to enhance recruitment (e.g., presentations at national and/or international meetings as the trial was conducted within a targeted demographic area). Furthermore, McDonald et al. [23] found that the only characteristics which were significantly related to the success of recruitment were being funded by the MRC, the intervention being based upon cancer treatment, and paying local recruitment co-ordinators, none of which applied to the Booster trial. This suggests a lack of generalisability for recruitment strategies regularly used in primary and secondary care to community-based trials.

**Table 3:** Comparison of the most commonly cited strategies and design features to maximise recruitment and Booster project examples

Feature/strategy	Booster project examples
Newsletters/mail-outs/flyers (to clinical staff and/or patients)	√ Mail-outs to participants
Regular visits/phone calls to wards/sites/practices	√Visits were made to local GP surgeries, health trainers/champions and one-stop health shops
Posters/information leaflets in clinics/wards/notes	√ Poster produced and displayed in GP surgeries
Inclusion criteria changed/protocol amended	√
Presentations to appropriate groups (e.g., at consults, meetings/community-based physiotherapists)	√ Presentations made to relevant organisations, NHS, local authority on becoming involved
Resource manual for site staff/trained staff in disease area/procedures being investigated/role-play exercises/study day/workshops for recruiters	√ Information provided to research assistants on recruitment
Advertisement/articles in newspapers/journals, radio interviews	√ Trial manager publicity in the local press, newspaper and radio
Presentations at national/international meetings	x
Employed extra staff	√ Administrative assistants hired
Investigators/recruiting staff meetings	√ Monthly meetings between trial manager and research assistants (in addition to monthly trial management group meetings), weekly email updates on recruitment
Training/information videos	x
Incentives for recruiters (e.g., prize draw)	√ Team rewarded at recruitment milestones
Trial material revised/simplified/customised for specific sites	√ Participant information sheet altered due to reflect added secondary recruitment pathways
Visits to centres by PIs/senior members of study group	√ Visits to local GP surgeries and to community venues
Repeated contact by phone/letter to individuals/sites	√ Reminder phone calls to participants
Increased/changed time points when information provided to potential participants	√ Additional mail-out conducted: extra letter to introduce main trial before screening call
Supportive statements from opinion leaders	x Supportive statements from current participants rejected by local research ethics committee
Quality of study team/multi-disciplinary team	√ Multi-disciplinary team from Sheffield Hallam University and University of Sheffield
Involvement of a Clinical Trials Unit	√ University of Sheffield Clinical Trials Unit, main collaborator in the trial
Trial manager	√ Dedicated project manager, appropriate cover arranged at a later date due to maternity leave
Local recruitment co-ordinators	x Centralised recruitment only
Feasibility work	√ Pilot study recruitment conducted
Peer-reviewed study protocol	√ Protocol publication
Simple study design	x Brief intervention and main intervention
Service user input	√ Expert elders attend design
Important research question with support of the clinical community	√ Support of public health, physical activity professionals, GPs and research community
Drug trial/intervention only available in study	√ Motivational interviewing not widely available in community
Appropriately funded	√ HTA funding secured for 3 years

*GP* general practitioner, *HTA* Health Technology Assessment, *NHS* National Health Service, *PI* principal investigator

Strategies were put in place to facilitate recruitment into the study. Table 4 lists these additional strategies and their impact from the anecdotal perspective of the RAs. A clear limitation is that the impact of these changes was not examined more objectively (e.g., there was not a mechanism put in place to determine recruitment from individual community groups or GP surgeries); therefore, a future recommendation is that researchers should use nested trial designs to ensure that the impact of manipulating the recruitment methods can be reliably investigated and that recruitment approach and/or setting can be assessed for each participant [24]. Initially, recruitment to the brief intervention was carried out exclusively via the mail-out. While some studies have found this approach to be successful [25], the use of multiple recruitment strategies is commonplace in physical activity studies [17]. In many cases, a pragmatic shift in recruitment strategies occurs, with additional approaches added as the study progresses, and recruitment fails to meet expectations. As in the Booster Study, Harland et al. [26] also set out to recruit sedentary 40–65-year-olds from socio-economically disadvantaged areas. They found that their initial approach of opportunistic recruitment via GP surgeries failed to yield sufficient numbers and subsequently added a postal recruitment pathway. The addition of indirect active approaches by GPs and community-based services did not appear to yield any extra recruits for the Booster Study, whereas Harland et al. [26] initially recruited a large number this way. These differences may be due to variation in how the opportunistic recruitment was carried out within the practice. The Booster Study investigators relied on the GPs or practice nurses to distribute recruitment packs, whereas Harland et al. [26] situated a researcher in the waiting room to approach all patients in the target age range. Recruitment to the main trial was then reliant on an active approach by the study team via telephone. Due to the heterogeneity in the recruitment methods and limitations in data available, it is difficult to draw any reliable conclusions on the most effective and cost-effective approach; therefore, we encourage researchers in future studies to include recruitment strategies in their cost-effectiveness analysis. The failure to recruit any patients via the indirect active approaches, which others have found to be effective [27], raises the question whether external organisations need to provide incentives for performing recruitment activities. Furthermore, reliance on active telephone recruitment to a large-scale study may have been an oversight. At the time, however, opportunities to use Internet-based technologies such as social media, which are now more commonly employed in health-based research [28], were limited. These modalities have the potential to be affordable in large-scale studies and could have reduced the high burden on researchers experienced during the Booster Study.

**Table 4:** Additional strategies implemented by the research team

Strategy	Outcome
Project manager attended an NIHR recruitment workshop.	No additional suitable strategies to use in the Booster Study were presented.
Additional recruitment pathway added. General practices were supplied with invitation packs to pass on to potential participants during consultations who may be eligible.	No reply cards were returned from participants who had received an invitation from their GP. There was no monitoring of the number of packs which were distributed to participants.
Time frame for recruitment was increased to maximise numbers, no extension required other research timescales were compounded.	Recruitment time was increased to ensure an extra mail-out could be conducted. It was understood that these late recruits would not provide 9-month follow-up data. Timescales at the end of the study (e.g. analysis, write-up) were condensed to accommodate this.
Text messaging and email contact was introduced where possible to check for DVD use during the brief intervention.	Managing incoming messages created new processes which were not managed effectively. The use of text messages and emails could not be used for screening calls, so a large amount of calls were still required. Strategy was phased out towards the end of recruitment.
Additional letter was sent to encourage those who had received the brief intervention and had increased their physical activity level to call in to complete main trial screening.	Some participants called in, although it did not greatly reduce the amount of calls to be made; it did, however, reduce the ‘cold call’ nature, as participants were made aware they would be contacted again.
Two administrative assistants were employed on a part-time basis.	Effective strategy to reduce administrative workload of RAs so that they had more time to concentrate on research-related activities. However, as administrative support was introduced at a later date, not all research activities where streamlined to use administrative support fully.
The final mail-out with smaller numbers and conducted whilst other project activities such as intervention delivery was less demanding. Hard copies of files were organised by the week when they needed to be called rather than their stage in the research. Tight deadlines for recruitment end were imposed.	It was clearer to the RAs what they needed to focus on and by a specific date. This reduced drift in the project timescales, and calls were made on time and received multiple attempts.
Participants were called the day before the appointment as a reminder, and opportunity to rearrange if the participant could no longer attend was provided.	It was felt that non-attendance still impacted the efficient use of room bookings and RA time.

*GP* general practitioner, *NIHR* National Institute for Health Research, *RA* research assistant

### Missed opportunities to understand recruitment from the pilot study

The study team noted a possible lack of representativeness between the participants in the pilot study and the recruitment pool for the full trial [18]. These concerns were justified, as the pilot study outperformed the full trial at most stages of the recruitment pathway. Socio-demographic analysis of the pilot sample revealed a disproportionate number of respondents were female (68.1 %), and, while this was in keeping with previous physical activity research [29], it did not match the demographics of the area. Recruitment into the full trial saw a more even split of male and female participants (53.9 % female), which, albeit surprising given the pilot data, is considered a strength of the trial recruitment process.

The people in the area chosen for the pilot study were known at the outset to be predominantly white and British. This area was chosen for two reasons—it is geographically isolated from the other target communities, and English was likely to be the main language spoken (at this stage, there was still the intention to offer the DVD in alternative languages for the full trial). While all areas identified for inclusion in the Booster Study were selected because they were deprived and at risk of significant health inequalities, the index of multiple deprivation indicated that two-thirds (38 of 57) of the neighbourhoods included in the full trial were more deprived than the pilot study area. This may have influenced the response rate, as previous researchers have identified lower response rates to research in deprived communities [29, 30]. Furthermore, there is generally an under-representation of individuals from lower socio-economic groups and ethnic minorities in physical activity interventions [31–33], which could have led to a greater dropout at each stage of study when comparing the pilot trial and the full trial.

The extent to which the lack of external validity in the pilot study impacted the recruitment in the main study was unforeseen. Despite the research team’s using the knowledge of local stakeholders on the trial management group, the pilot area might have been selected for reasons other than socio-demographic characteristics. Nevertheless, future studies should continue to engage local stakeholders in piloting studies and allow time for a thorough understanding of pilot study results. Furthermore, if researchers are concerned that the recruitment of their pilot study may not be representative of the full trial, it would be wise to consider community-specific recruitment strategies at the outset.

### Non-contactable participants

Almost half of those who registered interest for the brief intervention were non-contactable (*n* = 2462, 49.6 %). A further 840 participants (43.4 %) were non-contactable after the brief intervention to assess their eligibility for the RCT, despite attempts by the trial team to maintain contact during the brief intervention phase (e.g., text or phone call reminders to engage with the intervention). This pattern of recruitment loss remained consistent throughout the trial and represents a key reason why the Booster Study substantially under-recruited. It was suggested by the PCT and GP representatives on the research team that one of the key reasons that potential participants might be non-contactable is the highly transient nature of the populations in target areas. Several areas were known to have many newly arrived immigrants who subsequently moved to settle in other locations. In addition to this, residents of lower socio-economic areas are more likely to be in short-term rented accommodations. This could also explain the relatively high number of invitation letters that were ‘returned to sender’.

### Lack of provision for non-English speakers

The Booster project team anticipated a large response from people who speak English as a second language, and the funders were keen for the study to be as inclusive as possible. The study team was aware that this would potentially require bilingual RAs; therefore, during the set-up phase of the study, the study manager and PCT representative established the most prevalent languages in the target neighbourhoods. Although special dispensation was obtained from the university to actively recruit RAs who spoke Chinese, Urdu or Bangladeshi, there were no suitably skilled candidates with these language abilities.

The team received 162 requests for the brief intervention DVD in a language other than English. To retain as many prospective participants as possible, those who requested the DVD in another language were sent a second letter to offer an English version. This had limited success, so a large number of these participants did not receive the brief intervention. In comparison to other problems encountered in the study, the lack of DVDs in a second language might have had a relatively small influence on failing to recruit a sufficient number of participants. A consequence was that no non-English speakers were randomised, which reduces the generalisability of the findings to diverse communities. When targeting urban areas with ethnically diverse populations, such as Sheffield, receipt of only 162 requests for the intervention in another language from among over 70,000 invitation letters suggests that the language barriers or cultural differences could affect recruitment earlier than intervention delivery. This theory is supported by previous research into barriers to participation in health studies by ethnic minorities which suggested that non-personalised approaches, such as a standard mail-out, and a failure to provide project materials available in ethnic minorities’ languages act as barriers to participation [34]. Therefore, future research teams should consider whether recruitment strategies should be altered to suit the needs and preferences of ethnically diverse communities and to ensure that the team possesses the required language skills or contacts to support recruitment activities and deliver the intervention.

### Low rates of participant randomisation

Two hundred seventy-four individuals were eligible for the RCT but were not randomised. Participants who declined the offer to continue in the research most commonly said they were ‘not interested’ in the next stage of the study. Other reasons included lack of time due to work or family commitments, transport difficulties or not needing the additional support. All of these are commonly cited as reasons not to participate in research. The higher number (*n* = 113, 20.3 %) of cancellations and those recorded as ‘did not attend’ at baseline appointments than the non-attendance rates reported by Chinn et al. [29], who also targeted a similarly socially disadvantaged inner city area, seem to support this view. It should be noted, however, that the target population was also unique in that they had already participated in the first stage of the project, which was intended to help them become more active. The lack of recruitment into the RCT might have been influenced by their perception that they did not need any further help to stay active or that their expectations of becoming involved with the project may have already been fulfilled.

### Staffing/resource issues

A consultation with the RAs to discuss the recruitment issues at the end of the trial raised two key points regarding staffing and workload: (1) hiring six part-time staff, with the full-time equivalent of 3.0, did not provide sufficient cover for the research tasks, as staff had other commitments and projects which detracted from the time they were able to dedicate to the trial; and (2) the workload placed upon the RAs was unmanageable, and, despite the added administrative support and the staggered timing for the invitations to be sent, the sheer volume of calls and research activities was insurmountable.

The study was originally designed and funded with the intention of recruiting three full-time RAs. Local recruitment and redeployment policies, in conjunction with preferences of some RAs for part-time hours, led to the employment of six half-time RAs. With the benefit of hindsight, it would have been beneficial if some of the full-time RAs, whose time was split between the Booster Study and other projects, were actually allocated to the Booster Study full-time.

In terms of workload, mailing the study invitations detracted from the time available to perform other research tasks. The mail-outs were never intended to be one of the RAs’ tasks. Due to unforeseen circumstances and to the need to minimise a delay in recruitment, the research team agreed to undertake the mail-out with the additional support of two part-time administrative assistants. Nevertheless, this caused considerable problems with managing the competing interests of intervention delivery and study recruitment, which was further compounded when the number of invitations increased considerably.

Increasing the number of invitations is likely to have exacerbated the impact of operational issues of conducting the research, resulting in systems which were ineffective. The database system was also unable to prompt the research team when calls or follow-ups were due, so there was perhaps too much reliance on filing systems and spreadsheets to track the flow of participants through the study. When participants returned telephone calls, RAs had to trawl through the filing system whilst trying to ascertain which stage the participant had reached in the research, which could have appeared unprofessional and disorganised to the participants and likely contributed to delays in the research and recruitment process.

## Conclusions

The complex study design and the lack of external validity from the pilot sample to the full trial meant that recruitment was more difficult than anticipated for the Booster Study. This was compounded by a large workload and ineffective processes. That said, the research team supported the idea that increasing the number of invitation letters was necessary in response to the pilot study findings. In future, researchers should continue to thoroughly consider the implications of recruitment protocol changes and ensure that strategies are incorporated to limit any negative impact. Furthermore, adopting current or popular study design features and strategies in challenging areas for recruitment is not enough. Researchers need to be more innovative in their approach to recruitment and should always monitor the effectiveness of recruitment strategies during their research. Sharing recruitment information will support researchers in planning future studies and increase value for money for study funders, which may otherwise be left with studies that are unable to lead to confident conclusions regarding their primary research questions. With this in mind, it would be valuable for future researchers to consider adopting a formal qualitative review of their study recruitment, including in-depth exploration of the experiences of the research staff undertaking the trial so as to add to the evidence base.

### Lessons learnt

- Community-based RCTs in which investigators cannot use traditional care pathways for recruitment face challenges different from those in studies conducted with clinical populations.
- Researchers who rely on large-scale mail-outs may need to consider multiple recruitment pathways to suit the needs of diverse communities, and the early inclusion of such groups should be fully considered.
- Recruitment into an RCT should involve as few stages as possible to reduce the ‘funnel effect’. When this cannot be avoided, experienced researchers should provide guidance to ensure processes, workload and timescales are efficient and realistic to prevent the unnecessary loss of eligible participants.
- Complex recruitment designs place a greater importance on a thorough understanding of pilot study data to ensure that substantial changes to the recruitment strategies can be made if required. Time and cost should be written into research bids to ensure that changes and contingency plans can be added to the project.
- Get the basics right; staffing and data-handling processes should be time-effective and organised. Greater use of automated data management systems that prompt follow-up data capture points, for example, or that capture recruitment success via setting or approach would have greatly improved the experience and subsequent insights of the Booster Study.
- Those involved in the recruitment of participants for complex studies such as the Booster Study should be fully informed of the research question, research design and eligibility criteria, and appropriate resources and timescales should be applied to support the recruitment process.

## Abbreviations

CTRU: Clinical Trial Research Unit
EPHP: enhanced public health programme
GP: general practitioner
HTA: Health Technology Assessment
MRC: Medical Research Council
NHS: National Health Service
NICE: National Institute for Health and Care Excellence
NIHR: National Institute for Health Research
PCRN: primary care research network
PCT: primary care trust
PI: principal investigator
ScHARR: School of Health and Related Research, University of Sheffield
RA: research assistant
RCT: randomised controlled trial
START: systematic techniques for assisting recruitment to trials
